# Fabrication of supramolecular cyclodextrin–fullerene nonwovens by electrospinning

**DOI:** 10.3762/bjoc.15.10

**Published:** 2019-01-09

**Authors:** Hiroaki Yoshida, Ken Kikuta, Toshiyuki Kida

**Affiliations:** 1 Faculty of Textile Science and Technology, Shinshu University, 3-15-1 Tokida, Ueda, Nagano 386-8567, Japan; 2 Department of Applied Chemistry, Graduate School of Engineering, Osaka University, 2-1 Yamada-oka, Suita, Osaka 565-0871, Japan

**Keywords:** cyclodextrin, electrospinning, fullerene, nanofiber, supramolecular complex

## Abstract

Direct electrospinning of small molecules has great potential to fabricate a new class of fiber materials because this approach realizes the creation of various functional materials through the numerous molecular combinations. In this paper, we demonstrate a proof-of-concept to fabricate supramolecular fiber materials composed of cyclodextrin (CD)–fullerene inclusion complexes by electrospinning. Similar to the molecular state of fullerenes in solution, the resulting fibers include molecularly-dispersed fullerenes. We believe such a concept could be expanded to diverse host–guest complexes, opening up supramolecular solid materials science and engineering.

## Introduction

Fiber is a fundamental material that constitutes a variety of everyday items and supports the maintenance of life [1–3]. In the field of materials science and engineering, an underlying approach to produce fibers is polymer spinning. Polymer solutions or melts are generally used in the spinning process because their polymeric inter/intramolecular interactions and chain entanglements are supposed to work efficiently in the fiber formation process [4–5]. In 2006, Long et al. reported a unique and innovative approach to produce fibers via electrospinning [6]. They focused on the association behavior of surfactant molecules as a function of the solution concentration and demonstrated that phospholipids (lecithins) in nonaqueous media can be assembled into fibers/nonwovens by electrospinning. This result supports the notion that even small molecules (low-molecular weight compounds) with relatively weak inter/intramolecular interactions can be spun similar to the case of polymers. Since then, several small molecules, including gemini surfactants [7], diphenylalanine [8], cyclodextrin (CD)/CD derivatives [9–12], heteroditopic monomers [13], and self-assembling oligopeptides [14] have been successfully electrospun to produce continuous fibers.

Among these molecules, CD is a unique compound that can form fibers despite its low self-assembly features in solution [15]. We recently reported that 1,1,1,3,3,3-hexafluoroisopropanol (HFIP) is a suitable solvent for CDs, and a CD/HFIP solution can be facilely electrospun into fibers at a relatively low concentration of approximately 12.5 w/v % [11], which is comparable to the concentration for general polymer electrospinning. In addition to the academic potential of spinning small molecules, it may open new industrial applications. However, the functionalization of fiber materials composed of small molecules remains a challenging task. A reasonable approach to functionalize such fibers is to use host–guest inclusion complexes in CD electrospinning. To date, Uyar and Celebioglu have reported electrospinning of two different inclusion complexes: hydroxypropyl-β-CD (HP-β-CD)–tricosan [16] and HP-β-CD–azobenzene inclusion complexes [17]. Although such complexes are promising as an approach for fiber functionalization, the scope is limited to cases with 1:1 inclusion complexes and chemically modified CD.

Fullerenes have been widely studied in the fields of chemistry and materials science because they have attractive chemical structures and good electron acceptor abilities for free radical scavengers and solar cell applications [18–20]. A serious issue for practical applications of fullerenes is the poor solubility in most solvents. Various methods to improve the solubility have been demonstrated by coating the surface with surfactants or host molecules and introducing functional groups into the molecule directly. Among them, the formation of a 2:1 inclusion complex of γ-CD and C_60_ has been evaluated in various solvents such as water [21], toluene/water [22], DMSO [23], and DMF/water [24]. Although an impressive report that a 2:1 complex in water can be utilized as a homogeneous catalyst for nitrogen reduction under ambient conditions was published, the concentration of the complex in water is very low [25]. Thus, the development of γ-CD–C_60_ nonwovens by electrospinning might be useful as a novel inhomogeneous solid catalyst containing more C_60_.

**Figure 1:**
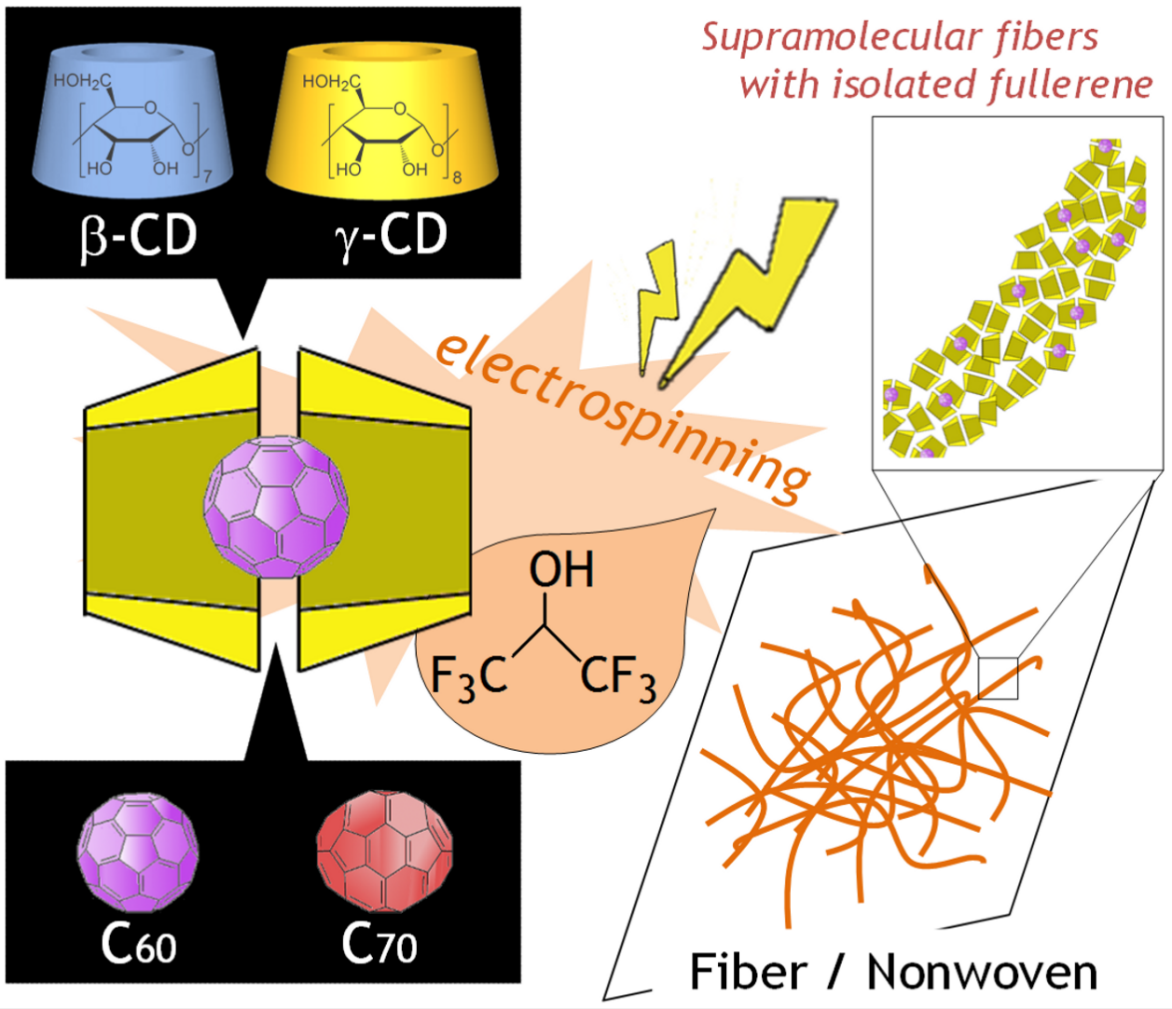
Schematic illustration of the fabrication of host–guest supramolecular fibers with molecularly dispersed fullerenes by direct CD electrospinning. In this study, β-CD or γ-CD is used as a host and C_60_ or C_70_ is used as a guest.

In this paper, we report the successful electrospinning of native CD–fullerene inclusion complexes in a HFIP solution to produce a new type of supramolecular fiber material. An advantage of our system compared with the previous technique [10] is that only 12.5–20 w/v % CD/HFIP solution is required. This realizes easy handling of inclusion complexation with guest molecules as well as electrospinning due to the much lower viscosity of the CD/HFIP solution. The formation of a 2:1 inclusion complex should not affect the solution properties (e.g., viscosity and solubility), but should provide electrospinning parameters similar to the case without the guest because the guest molecule is isolated from the solvent molecules by two γ-CD molecules. This is in contrast with the part of the guest molecule uncovered by γ-CD which may interact with the solvent molecules in the case of a 1:1 inclusion complex. Moreover, isolation of fullerene by γ-CD motivated us to fabricate CD–fullerene inclusion complex fiber materials with molecularly dispersed fullerenes (Figure 1).

## Results and Discussion

A representative example of a CD–fullerene inclusion complex is the combination of γ-CD and C_60_ [21–24]. Although the formation of the γ-CD–C_60_ complexes has been reported in both aqueous and organic media, including water [21], toluene/water [22], DMSO [23], and DMF/water [24], electrospinning is difficult due to the low solvent volatility. Therefore, an alternative solvent with a higher volatility must be explored to form γ-CD–C_60_ complexes.

**Figure 2:**
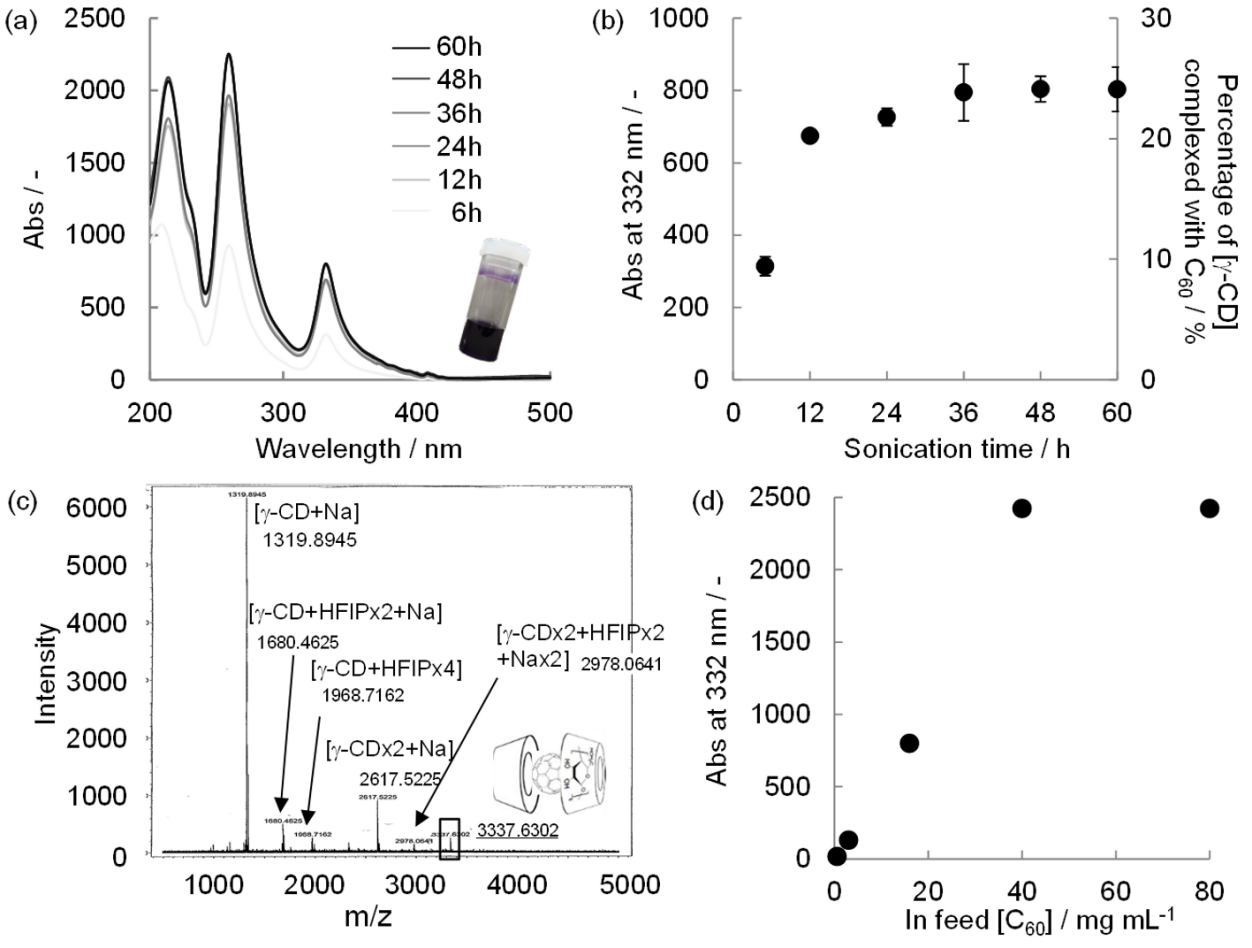
Formation of a γ-CD–C_60_ inclusion complex in HFIP under sonication for 60 h. (a) UV–vis absorption spectra of the solutions. Inserted photograph shows a typical, purple-colored complex solution. (b) UV–vis absorbance at 332 nm (left y-axis) and the percentage of γ-CD complexed with C_60_ (right y-axis) with sonication time (*n* = 3). C_60_ (16 mg mL^−1^) was added to 15 w/v % of γ-CD/HFIP. (c) ESI-mass spectrum of a typical complex solution shows the formation of the 2:1 complex by the peak at 3337.6302. (d) UV–vis absorbance of the final solution vs feed C_60_ amount (0.6, 3.2, 16, 40, 80 mg mL^−1^, *n* = 3).

Our electrospinning system employs a highly volatile solvent, HFIP. We initially examined γ-CD–C_60_ complex formation in HFIP. C_60_ (16 mg/mL, pre-ground by an agate mortar) was added into 15 w/v % γ-CD/HFIP and kept under sonication for a few days. After removing the residual C_60_ by filtration, the obtained purple solution shows the UV–vis absorption peaks (214, 260, 332, and 408 nm, Figure 2a). The spectrum agrees well with those of C_60_ in toluene or cyclohexane [21]. The absorption peaks increase as the sonication time increases until an equilibrium is reached after 24–36 h (Figure 2b and Figure S1 in Supporting Information File 1). Although complex formation occurs without sonication, the color of the obtained solution was fairly weak (data not shown). Shortening of the time to reach equilibrium in the γ-CD–C_60_ inclusion complex formation was reported by downsizing C_60_ with bowl milling [26] and high-speed vibration milling [27]. We confirmed that a simple grinding process by an agate mortar is sufficient for the HFIP system (Figure S2 in Supporting Information File 1).

ESI mass spectrometry of the purple solution indicates the presence of the γ-CD–C_60_ (2:1) inclusion complex, γ-CD dimer, and γ-CD monomer in HFIP. Interestingly the γ-CD–C_60_ (1:1) inclusion complex, which is the supposed intermediate, is not detected (Figure 2c in Supporting Information File 1). Therefore, we considered that all C_60_ molecules in the solution should be present as the 2:1 complex. The percentage of γ-CD complexed with C_60_ is estimated to be approximately 25% at the equilibrium state (Figure 2b**)**, where the extinction coefficient of the 2:1 complex in HFIP was calculated from the UV–vis absorption spectra of the mixed solutions of γ-CD/HFIP and C_60_/toluene (Figure S3, Table S1 in Supporting Information File 1). Additionally, the concentration of the 2:1 complex (or C_60_) is 1.5 × 10^−2^ M, which is ten times higher than the previously reported maximum concentration (1.4 × 10^−3^ M) [24]. These values are further increased up to 75% (4.5 × 10^−2^ M) by controlling the equilibrium. That is, simply by increasing the feed of C_60_ up to 40 mg/mL can control the equilibrium (Figure 2d). However, such a highly concentrated C_60_ solution is unstable and the solution changes to dark brown after a few days.

In the electrospinning of small molecules, controlling intermolecular association in solution is essential. Association is predicted from the relationship of the solution viscosity and concentration [6,9,11]. As reported previously, the increased rate of viscosity with the concentration in γ-CD/HFIP solution clearly becomes larger at 10–15 w/v %, indicating intermolecular associations of γ-CD molecules in HFIP [11].

**Figure 3:**
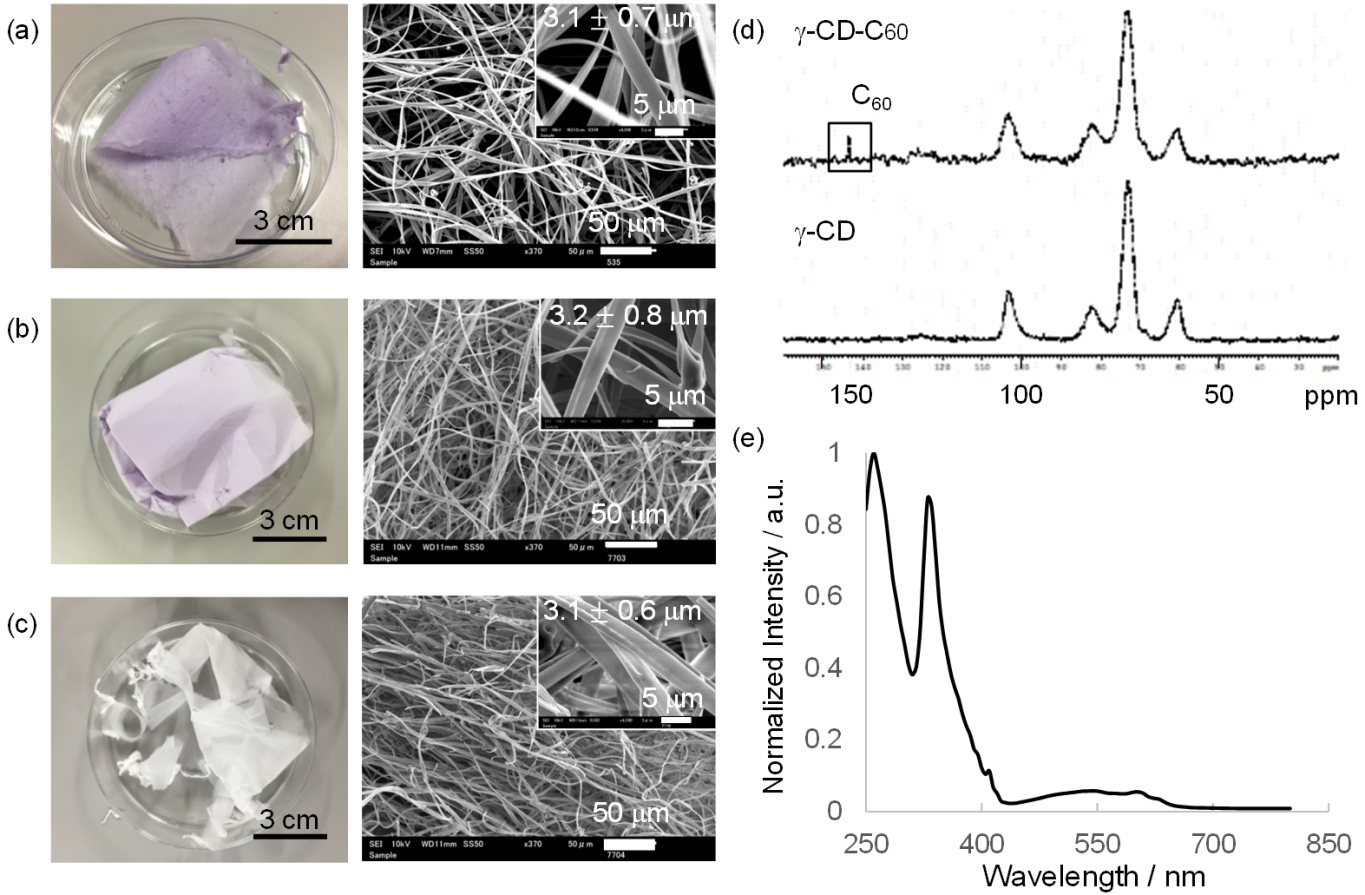
Fabrication of γ-CD–C_60_ inclusion complex nonwovens by electrospinning. Photographs and SEM images of (a) γ-CD–C_60_ nonwoven ([C_60_] = 1.5 × 10^−2^ M), (b) γ-CD–C_60_ nonwoven ([C_60_] = 2.6 × 10^−3^ M), and (c) γ-CD nonwoven as a control. Fiber diameter is calculated from SEM images of three different samples (*n* = 100). (d) ^13^C CP/MAS NMR spectrum and (e) height-normalized UV–vis reflectance spectrum of γ-CD–C_60_ nonwoven.

Interestingly, no significant viscosity differences are observed after C_60_ addition into γ-CD/HFIP solution (Figure S4 in Supporting Information File 1). This is important from the viewpoint that the solution properties are governed by the intermolecular interactions between γ-CD, even in a complex solution containing C_60_. Direct electrospinning of the γ-CD–C_60_ complex was performed at a C_60_ concentration of 1.5 × 10^−2^ or 2.6 × 10^−3^ M. Fiber formation is observed over a wide range of accelerating voltages (10–30 kV), distances between electrodes (5–15 cm), and flow rates (0.6–15 mL/hour). The most homogeneous microfibers during long-time electrospinning are formed at the optimized parameters of 15 kV, 3 mL/hour, 10 cm (Figures S5–S7 in Supporting Information File 1). The γ-CD–C_60_ nonwovens obtained after 1 h of electrospinning surprisingly shows a purple color and the color strength is clearly related with the C_60_ concentration in the complex solution used for electrospinning. This is completely different from the white γ-CD nonwovens without C_60_ (Figure 3a–c). SEM observations clearly suggest that the microstructure of the samples are the almost same regardless of the incorporation of C_60_, and the fiber diameter is approximately 3 μm. This may be because all solutions have similar solution properties.

To clarify the inner structure of the γ-CD–C_60_ fibers, solid-state ^13^C NMR and UV–vis diffuse reflectance spectroscopies were performed. As shown in Figure 3d, solid-state ^13^C NMR clarifies the presence of C_60_ in the fibers, but does not provide additional information. Solid-state UV–vis reflectance spectroscopy clearly suggests reflectance peaks at 260, 330, and 410 nm. This spectrum is almost the same as those of the γ-CD–C_60_ complex solutions (Figure 3e). In addition, the UV–vis intensity increases with increasing C_60_ concentrations.

This technique can disperse C_60_ easily to aggregate in a γ-CD fiber matrix, producing supramolecular host–guest solid fiber materials with molecularly dispersed C_60_. However, it is not easy to demonstrate the presence of an inclusion complex structure in solvent-free supramolecular solid materials at the molecular level. Although most small molecules, including CD, generally give crystalline solids after simple casting or vacuum drying [11,28–29], electrospinning of small molecules typically provides structurally amorphous fiber materials consistently [9,11]. In this work, we expected that the incorporation of C_60_ into γ-CD nanofibers helps with the regular arrangement of γ-CD molecules, but γ-CD–C_60_ microfiber materials do not also show a specific XRD pattern (Figure S8 in Supporting Information File 1).

Because the red photoluminescence of C_60_ is useful for a bioimaging applications [30–31], the electrospun fibers were measured with confocal laser scanning microscopy (CLSM). Interestingly, a uniform red color distribution is observed (Figure S9, in Supporting Information File 1), indicating the presence of C_60_ throughout the fibers. Two additional experiments were performed to confirm the presence of the CD–C_60_ inclusion complex indirectly. One investigated C_60_ extraction by toluene washing of the nonwovens. Toluene is a good solvent for C_60_, but UV–vis absorption peaks assignable to C_60_ are not detected even after the nonwovens were stored in toluene for three days (Figure S10 in Supporting Information File 1). The other aimed examined the solution of the nonwovens re-dissolved with HFIP. The resulting purple solution clearly provides the same UV–vis absorption and ESI mass results as the original solution (data not shown). Taken all together, it is reasonable to consider that C_60_ is an inclusion complex with γ-CD even in solid fibers.

To expand the applicability of CD–fullerene inclusion complexes, variations of CD/fullerene and embedding into a polymer matrix were explored. Other examples of CD–fullerene pairs to form similar 2:1 inclusion complexes are β-CD–C_60_ [32] and γ-CD–C_70_ [33]. However, such combinations are unlikely to form inclusion complexes in solution compared with that of γ-CD–C_60_ due to the mismatched size of CD and fullerene.

**Figure 4:**
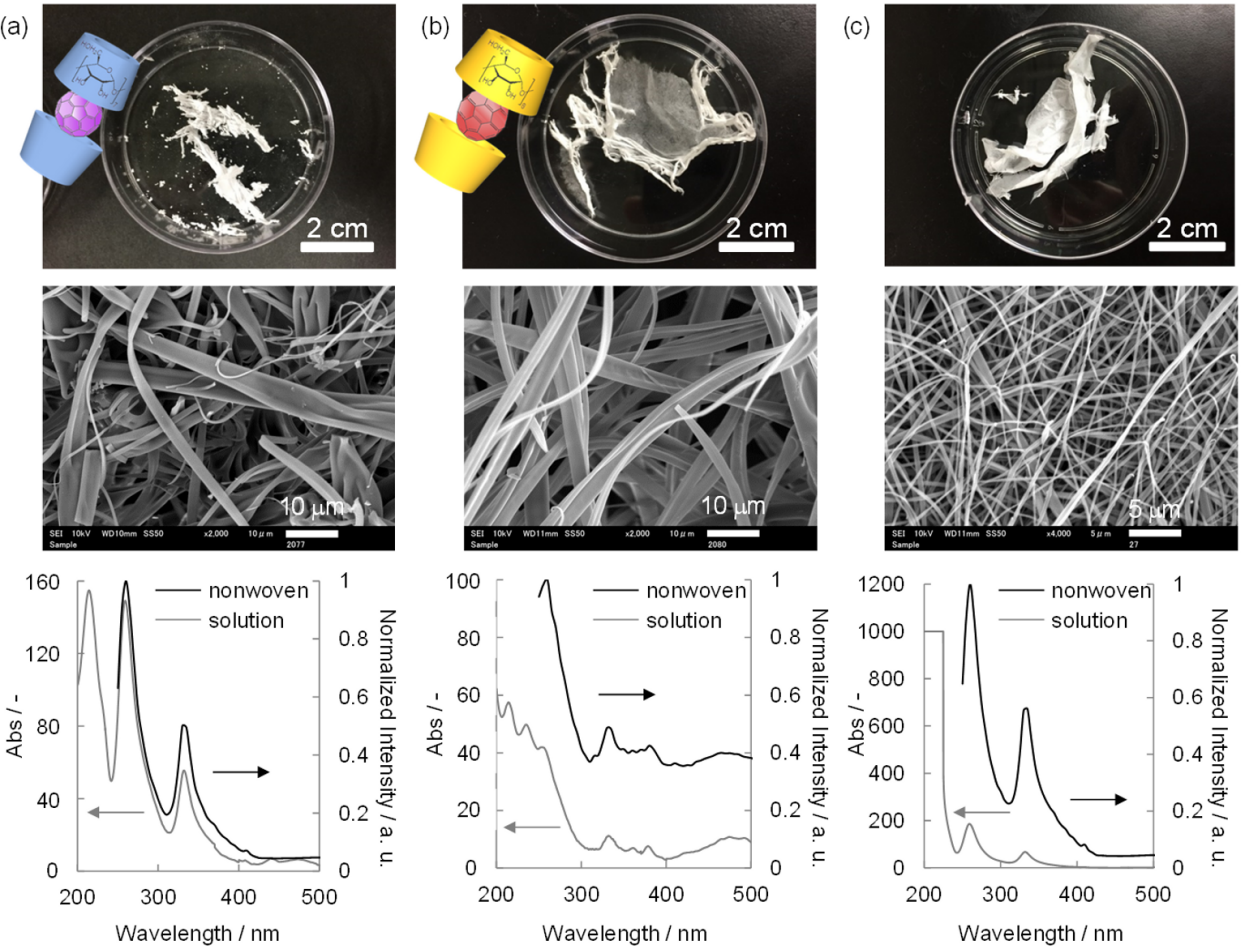
Extended variation of CD–fullerene inclusion complex to fabricate supramolecular solid functional fibers by electrospinning. Photographs, SEM images, and UV–vis absorption (solutions)/diffuse reflectance (nonwovens) spectra of (a) β-CD–C_60_ and (b) γ-CD–C_70_. (c) Embedding of molecularly-dispersed C_60_ into a gelatin matrix by simply mixing gelatin and the γ-CD–C_60_ complex.

The formation of both inclusion complexes and the subsequent electrospinning was performed in the same manner as conducted previously (Figure 4a,b and Figure S11 in Supporting Information File 1). The obtained β-CD–C_60_ solution is pale brown, and the UV–vis absorption peaks (214, 259, 332, and 408 nm) are consistent with that of the previous report [32]. The UV–vis intensity is 100 times smaller, but is estimated to have the same extinction coefficient as the γ-CD–C_60_ solution, possibly due to the insufficient interaction between β-CD and C_60_. The electrospun fibers have an inhomogeneous diameter of 1.5 ± 1.0 μm and show similar UV–vis reflectance peaks (around 255 and 330 nm). In the case of γ-CD–C_70_, the solution is pale dark purple with UV–vis absorption peaks (214, 235, 254, 332, 361, 378, and 474 nm) assignable to C_70_ [33], and electrospun fibers with a diameter of 2.0 ± 0.74 μm with UV–vis diffuse reflectance peaks (around 260, 330, 380, and 475 nm) are observed. These results clearly suggest the successful preparation of β-CD (γ-CD) fiber materials with molecularly dispersed C_60_ (C_70_).

The preparation of hybrid materials of a polymer and a CD–fullerene inclusion complex might be interesting to enhance material integrity and extend applications (Figure 4c and Figure S11 in Supporting Information File 1). Herein a biocompatible polymer (gelatin) was chosen as the polymer matrix because the polymer has good solubility in HFIP [34]. The solution was simply prepared by mixing gelatin/HFIP and γ-CD–C_60_/HFIP. The obtained solution maintains the same UV–vis absorption (259 and 332 nm). Electrospinning in the same manner produces a slightly purple nonwoven composed of homogeneous nanofibers with a diameter of 0.34 ± 0.18 μm and with a reasonable UV–vis diffuse reflectance (around 265 and 335 nm). These results suggest that the mixing of the complex into the polymer matrix does not affect the chemical structure of the inclusion complex or the electrospinning parameters of gelatin.

## Conclusion

In conclusion, functionalized CD fiber materials are successfully prepared by direct electrospinning of CD–fullerene (γ-CD–C_60_, β-CD–C_60_ and γ-CD–C_70_) inclusion complexes. The formation of such inclusion complexes in HFIP does not change the solution properties. Consequently, similar electrospinning parameters can be applied despite the incorporation of fullerene. The resulting nonwovens show similar colors to those of the solutions. UV–vis diffuse reflectance spectroscopy suggests that the C_60_ molecules are isolated in the fibers at the molecular level. We believe that inclusion complexation with various guest molecules will fabricate a wider range of functional CD fiber materials containing isolated guest molecules by electrospinning.

## Experimental

β-CD (98%, Wako Pure Chemical Industries Ltd., Japan), γ-CD (98%, Tokyo Chemical Industry Co., Ltd., Japan), HFIP (99%, Fluorochem Ltd., UK), C_60_ (99.5%, Filgen Inc.), C_70_ (99%, Filgen Inc.), gelatin (Wako Pure Chemical Industries Ltd., Japan) were used in this study. Fullerenes were ground by an agate mortar for 10 min before use.

CD was dissolved in HFIP under sonication at the pre-determined concentration (typically, 23 w/v % for β-CD and 15 w/v % for γ-CD). Fullerene was added into the solution at the pre-determined concentration and sonicated for a few days. The obtained solution was purified with a syringe filter (0.45 μm) to remove the remaining fullerene solids. The gelatin/γ-CD–C_60_ solution was prepared by mixing gelatin/HFIP (9.4 w/v %) with γ-CD–C_60_/HFIP ([γ-CD] = 15 w/v %, [C_60_] = 1.5 × 10^−2^ M) at a ratio of 10:1 w/v %. The resulting solution was evaluated by UV–vis spectroscopy (V-730, JASCO, Japan), ESI mass spectroscopy (Autoflex III, Bruker), and small sample viscometry (m-VROC^TM^, RheoSense, USA).

γ-CD/HFIP (15 w/v %; 500 μL) and C_60_/toluene (0, 0.14, 0.29, 0.43, 0.58, 0.72 mM; 25 μL) were mixed and measured by UV–vis spectrometry. The calibration curve was prepared from the absorbance at 332 nm, and the molar extinction coefficient was calculated from the Lambert–Beer law.

Electrospinning was performed with a Nanofiber Electrospinning Unit (Kato Tech, Japan). The solution was pumped through a single-use blunt-end 18-gauge cannula at a flow rate of 0.6, 3, or 15 mL/hour, and the collection distance between the cannula and the rotating drum target (diameter: 10 cm, width: 33 cm) was 5–15 cm. The drum substrate was covered with aluminum foil and rotated at a rate of 2.0 m/min during the electrospinning of the solutions. A voltage of 10–30 kV was applied between the cannula and the substrate. Nonwovens were prepared after 1 hour of electrospinning. The obtained nonwovens were evaluated by SEM (JSM-6010LA, JEOL, Japan), UV–vis diffuse reflectance spectrum (V-670 spectrometer with an integration sphere attachment, JASCO), ^13^C CP/MAS NMR spectrum (Bruker, USA), X-ray diffraction (MiniFlex 300, Rigaku, Japan), and CLSM (FLUOVIEW FV1000, Olympus, Japan). The optimized electrospinning parameters were: (γ-CD–C_60_) voltage: 15 kV, distance between electrodes: 10 cm, flow rate: 3 mL/h; (β-CD–C_60_) voltage: 25 kV, distance between electrodes: 10 cm, flow rate: 1.8 mL/h; (γ-CD–C_70_) voltage: 25 kV, distance between electrodes: 10 cm, flow rate: 3 mL/h; (gelatin/γ-CD–C_60_) voltage: 10 kV, distance between electrodes: 15 cm, flow rate: 1 mL/h.

## Supporting Information

File 1 UV–vis and viscosity measurements of the spinning solutions, electrospinning at various parameters, and XRD patterns of the prepared nonwovens.
