# Supplementary material for: Fabrication of supramolecular cyclodextrin–fullerene nonwovens by electrospinning
Source: Beilstein J Org Chem. 2019 Jan 9;15:89–95. doi: 10.3762/bjoc.15.10 (PMC6334797; doi:10.3762/bjoc.15.10)
Supplement: File 1 — UV–vis and viscosity measurements of the spinning solutions, electrospinning at various parameters, and XRD patterns of the prepared nonwovens. [file Beilstein_J_Org_Chem-15-89-s001.pdf]

BEILSTEIN JOURNAL OF ORGANIC CHEMISTRY

Supporting Information

for

Fabrication of supramolecular cyclodextrin–fullerene nonwovens by electrospinning

Hiroaki Yoshida, Ken Kikuta and Toshiyuki Kida

Beilstein J. Org. Chem. **2019,** *15,* 89–95. doi:10.3762/bjoc.15.10

UV–vis and viscosity measurements of the spinning solutions, electrospinning at various parameters, and XRD patterns of the prepared nonwovens

License and Terms: This is a supporting information file under the terms of the Creative Commons Attribution License (http://creativecommons.org/licenses/by/4.0). Please note that the reuse, redistribution and reproduction in particular requires that the authors and source are credited.
The license is subject to the *Beilstein Journal of Organic Chemistry* terms and conditions: (https://www.beilstein-journals.org/bjoc)

Contents

1. (Figure S1) Change of the complex solution color with sonication time **S2**

2. (Figure S2) The effect of C_{60} grinding for preparation of γ-CD–C_{60} solution **S2**

3. (Figure S3, Table S1) Calculation of extinction coefficient of the γ-CD–C_{60} complex in HFIP **S3**

4. (Figure S4) Difference in solution viscosity between γ-CD/HFIP with/without C_{60} **S4**

5. (Figure S5-7) Investigation on detailed electrospinning parameters for γ-CD□C_{60} solution **S5**

6. (Figure S8) XRD patterns of γ-CD–C_{60} nonwovens prepared by electrospinning **S8**

7. (Figure S9) CLSM observation of γ-CD–C_{60} fibers **S8**

8. (Figure S10) Investigation on C_{60} extraction from the nonwovens by toluene washing **S9**

9. (Figure S11) XRD patterns of β-CD–C_{60}, γ-CD–C_{70}, and gelatin/γ-CD–C_{60} nonwovens prepared by electrospinning **S9**

1. (Figure S1) Change of the complex solution color with sonication time

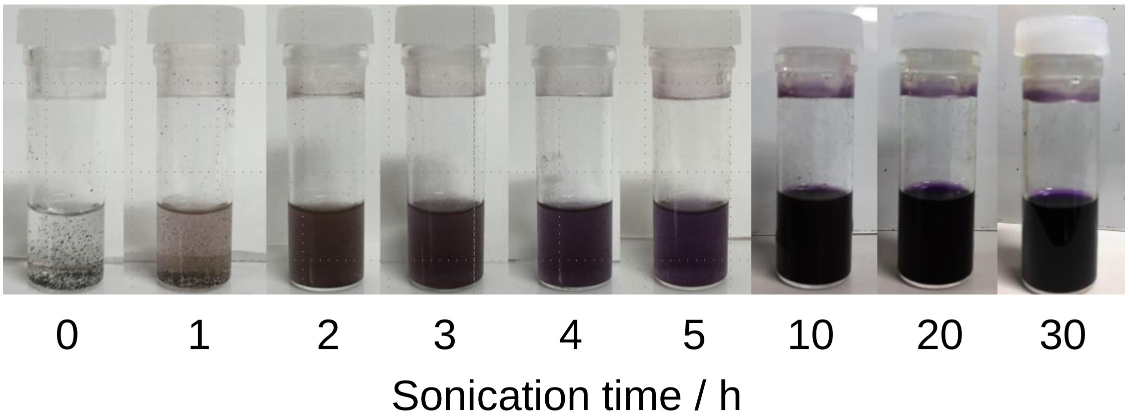

Figure S1. Change of the complex solution color with sonication time. These solutions still contains residual C60.

2. (Figure S2) The effect of C_{60} grinding for preparation of γ-CD–C_{60} solution

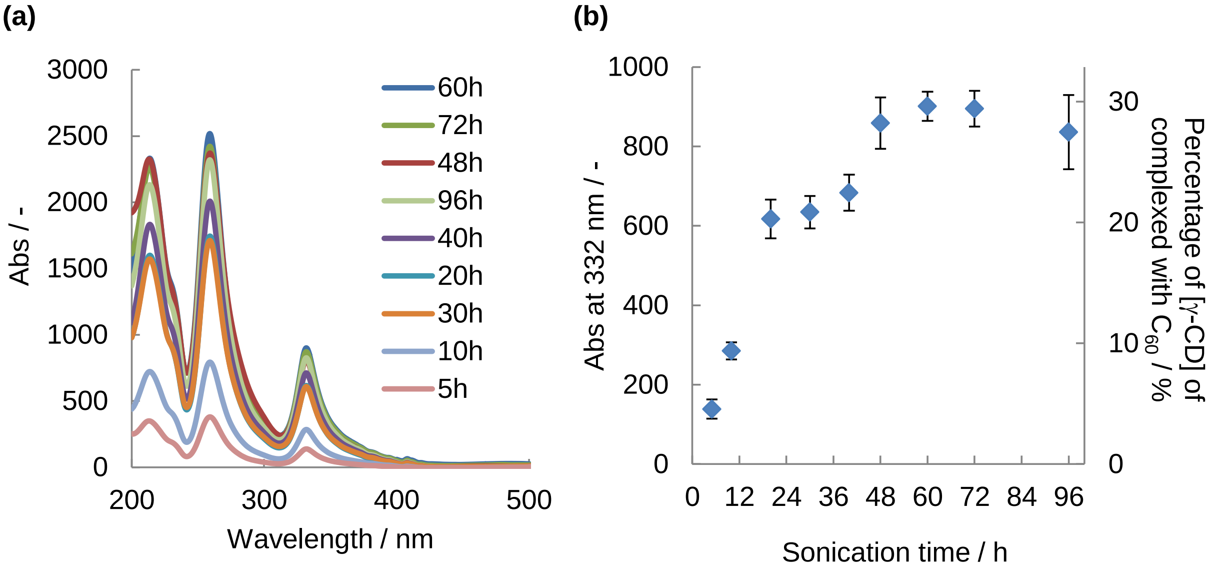

Figure S2. (a) UV–vis absorption spectra of 15 w/v % of γ-CD/HFIP containing 16 mg mL^{-1} of C_{60} (without C_{60} grinding before use) under sonication, and (b) the absorbance at 332 nm (left *y*-axis) and the percentage of γ-CD complexed with C_{60} (right *y*-axis) with sonication time (n = 3).

3. (Figure S3, Table S1) Calculation of extinction coefficient of the γ-CD–C_{60} complex in HFIP

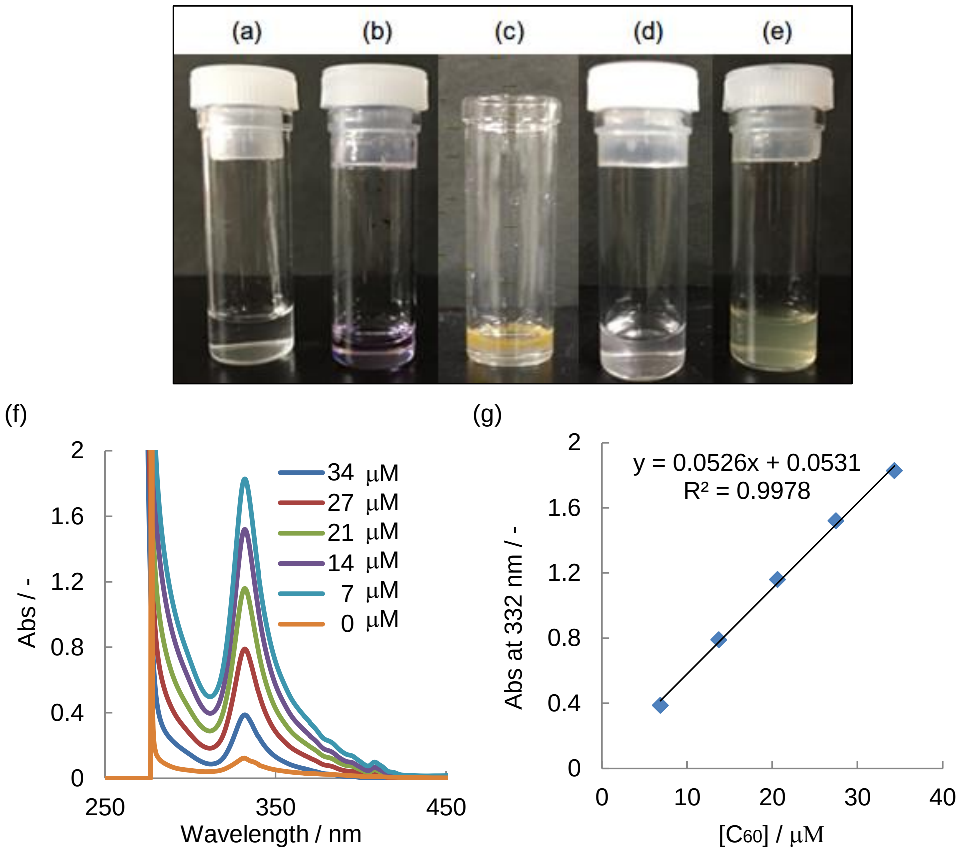

Figure S3: Photographs of (a) 15 w/v % γ-CD/HFIP, (b) 3.3 mM C_{60}/toluene, mixture of 15 w/v % γ-CD/HFIP and 3.3 mM C_{60}/toluene solution at the ratio of 20:1 v/v % (c) immediately after mixing and (d) after less than 10s, and (e) mixture of HFIP and 3.3 mM C_{60}/toluene solution at the ratio of 20:1 (v/v), which suggests that the presence of γ-CD is important to dissolve C_{60} in HFIP. (f) UV–vis spectra of the mixed solutions of 15 w/v % γ-CD/HFIP and various concentration of C_{60}/toluene (0, 7, 14, 21, 27, 34 mM) at the ratio of 20:1 (v/v). (g) Abs at 332 nm vs [C_{60}] (n = 3). Extinction coefficient of the γ-CD–C_{60} complex in HFIP was calculated to be 52,550 ± 2565 from the slope.

Table S1. Comparison of extinction coefficient of C_{60} and γ-CD-C_{60} solutions.

Compound	Solvent	Extinction Coefficient	Reference
C_{60}	Toluene	5.5×10^4 M^{-1} cm^{-1}	1
C_{60}	n-Hexane	5.3×10^4 M^{-1} cm^{-1}	2
C_{60}	Cyclohexane	5.2×10^4 M^{-1} cm^{-1}	3
γ-CD-C_{60}	Water	1.1×10^4 M^{-1} cm^{-1}	4
γ-CD-C_{60}	HFIP	5.3×10^4 M^{-1} cm^{-1}	This work

1) R. V. Bemasson, E. Bienvenue, M. Dellinger, S. Leach, P. Seta, *J. Phys. Chem.* **1994**, *98*, 3492.
2) S. Sawamura, N. Fujita, *Carbon* **2007**, *45*, 965.
3) K. Komatsu, K. Fujiwara, Y. Murata, T. Braun, *J. Chem. Soc., Perkin Trans* **1999**, *1*, 2963.
4) K. I. Priyadarsini, H. Mohan, A. K. Tyagi, J. P. Mittal, *J. Phys. Chem.* **1994**, *98*, 4756.

4. (Figure S4) Difference in solution viscosity between γ-CD/HFIP with/without C_{60}

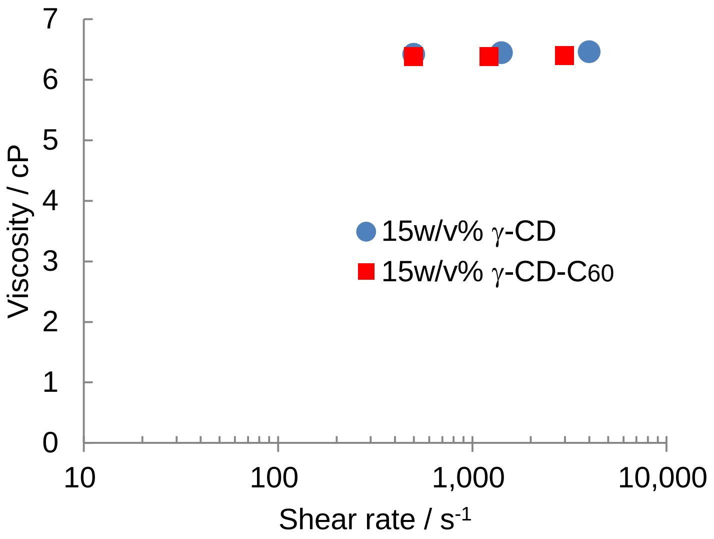

Figure S4. Change of solution viscosity by complexation with C_{60} in 15 w/v % γ-CD/HFIP solution. We did not find significant difference in viscosity between the two solutions.

5. (Figure S5-7) Investigation on detailed electrospinning parameters for γ-CD–C_{60} solution

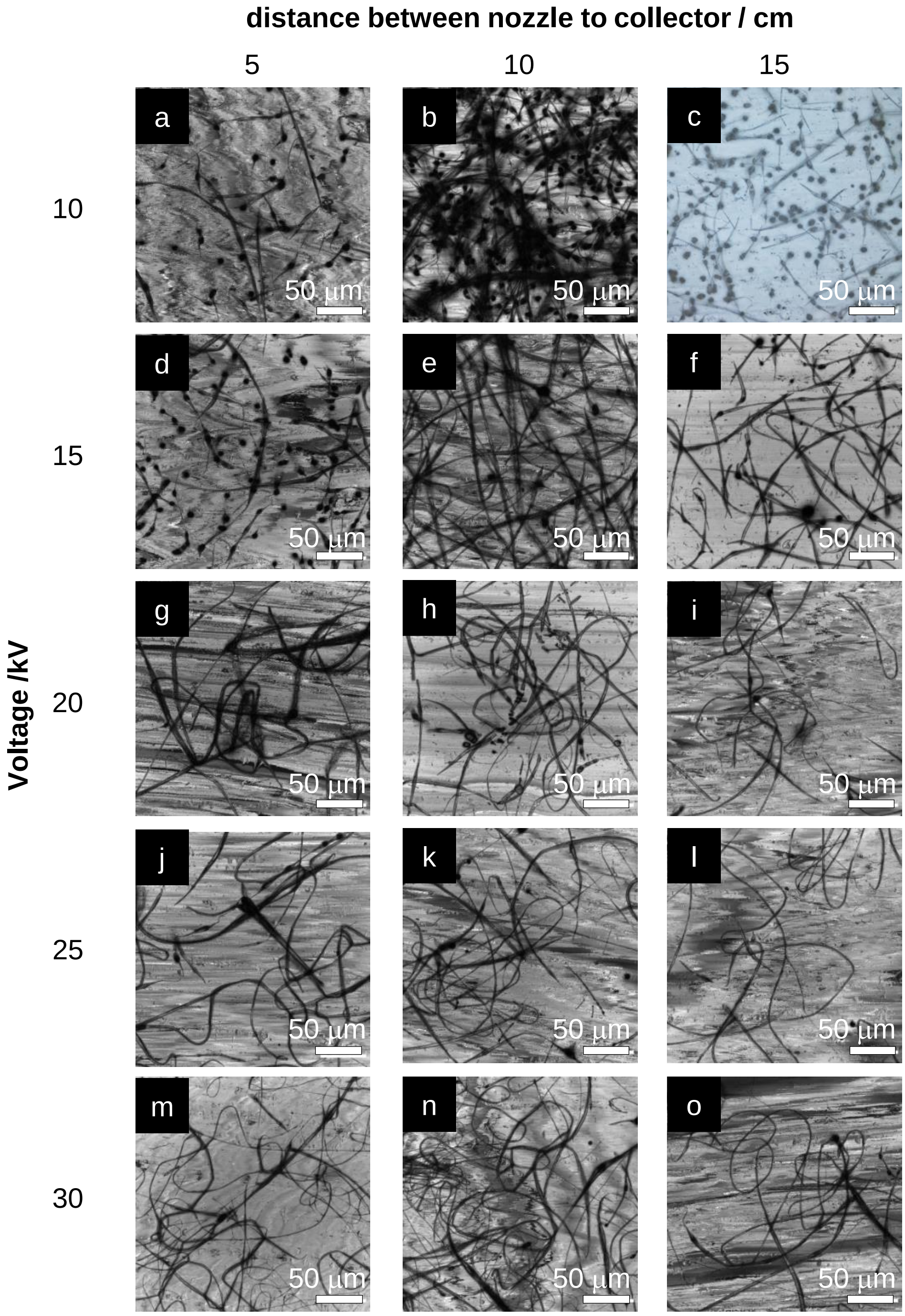

Figure S5. Electrospinning of 15 w/v % γ-CD/HFIP containing 1.5×10^{-2} M of C_{60}. Flow rate was 0.6 mL/h.

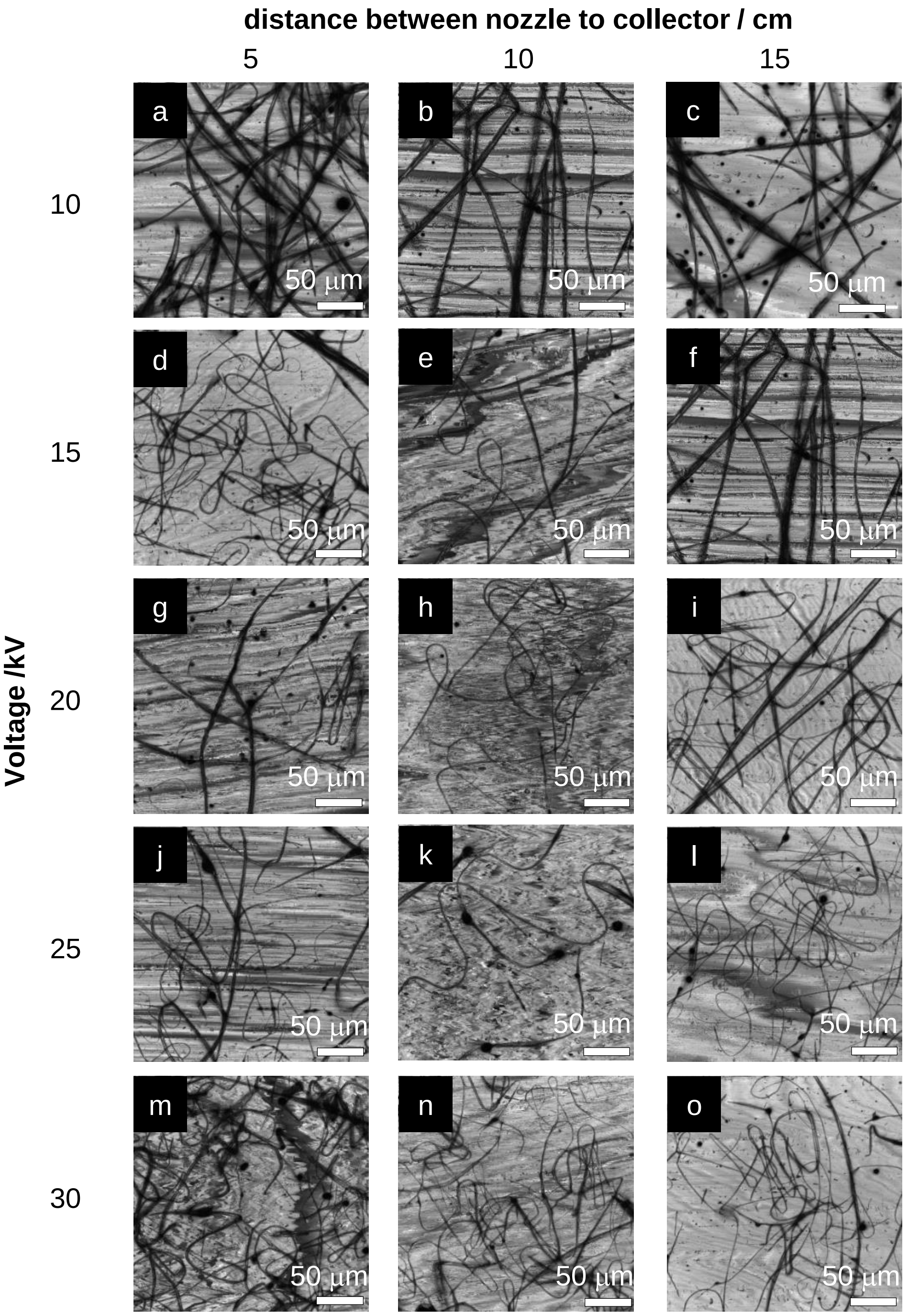

Figure S6. Electrospinning of 15 w/v % γ-CD/HFIP containing 1.5 × 10^{-2} M of C_{60}. Flow rate was 3 mL/h.

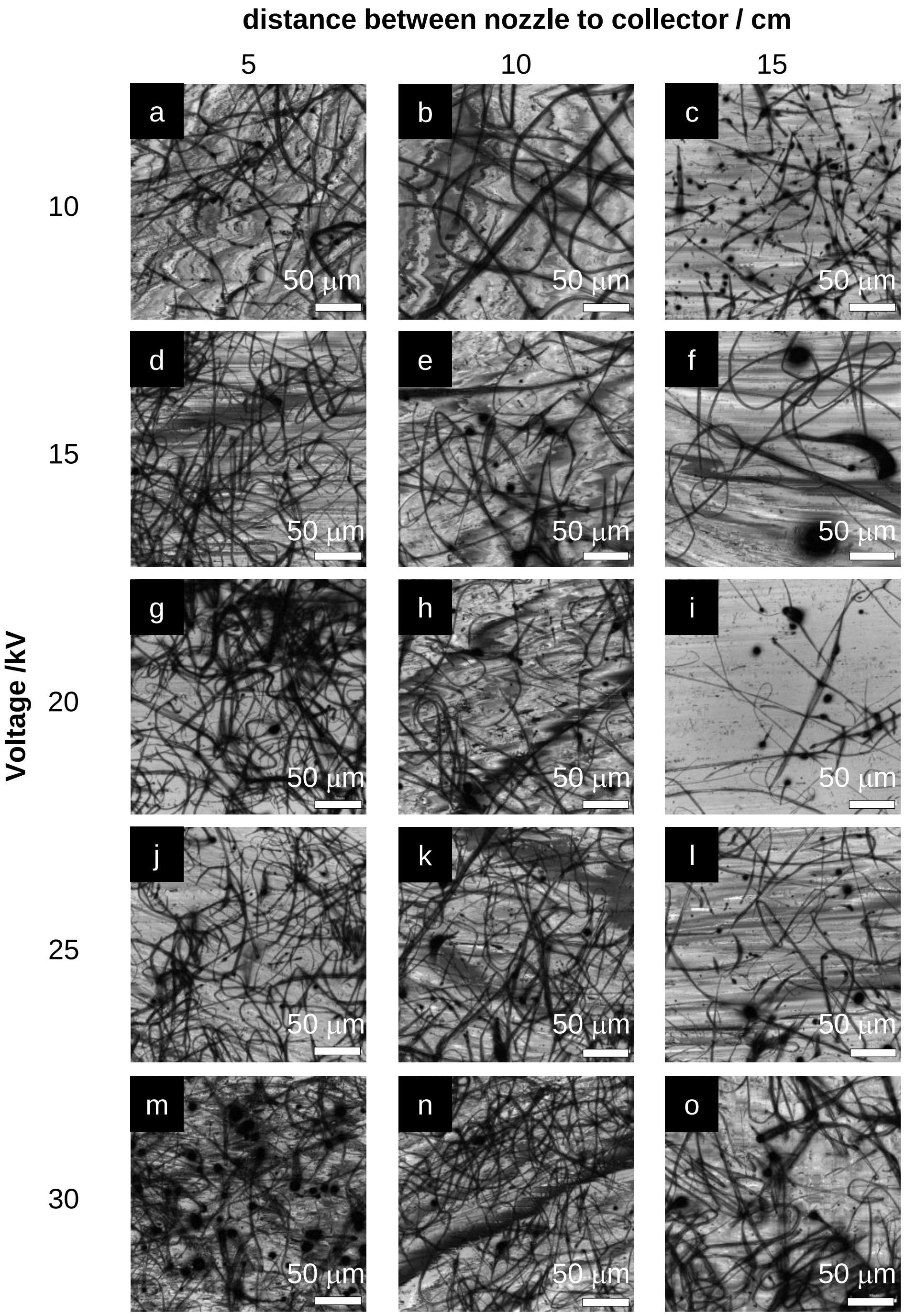

Figure S7. Electrospinning of 15 w/v % γ-CD/HFIP containing 1.5×10^{-2} M of C_{60}. Flow rate was 15 mL/h.

6. (Figure S8) XRD patterns of γ-CD–C_{60} nonwovens prepared by electrospinning

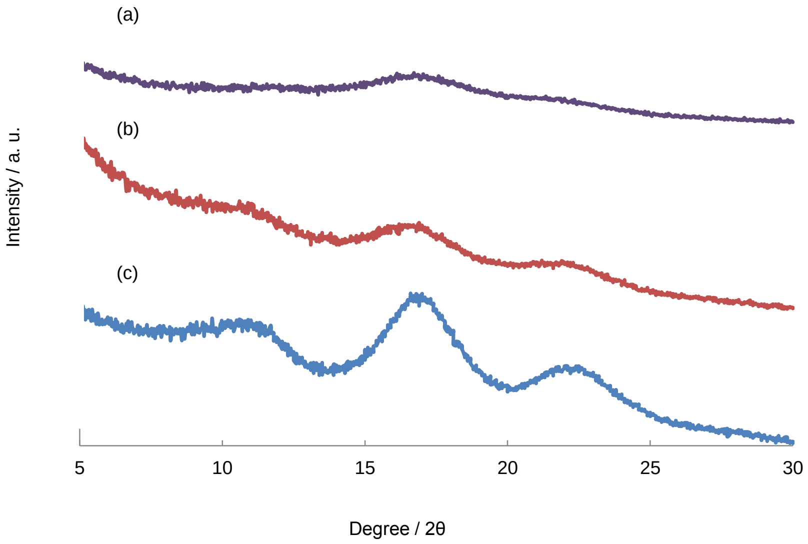

Figure S8. XRD patterns of nonwovens prepared by electrospinning of (a) γ-CD–C_{60}/HFIP ([C_{60}] = 1.5 × 10^{-2} M), (b) γ-CD–C_{60}/HFIP ([C_{60}] = 2.6 × 10^{-3} M), and (c) γ-CD/HFIP. [γ-CD] = 15 w/v %.

7. (Figure S9) CLSM observation of γ-CD–C_{60} fibers

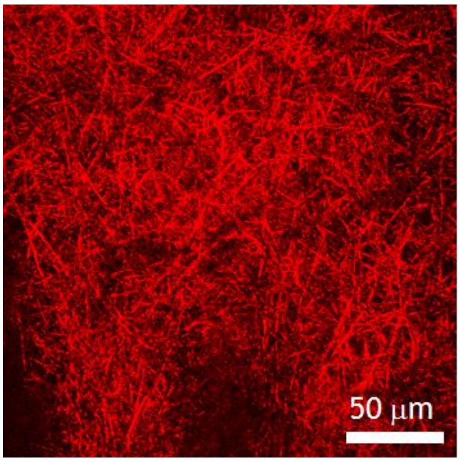

Figure S9. CLSM observation of nonwovens prepared by electrospinning of γ-CD–C_{60}/HFIP ([C_{60}] = 2.6 × 10^{-3} M). The image was obtained with a FluoView FV1000 (Olympus, Japan) equipped with the fluorescence filter (ex. 559 nm / em. 647 nm)

8. (Figure S10) Investigation on C_{60} extraction from the nonwovens by toluene washing

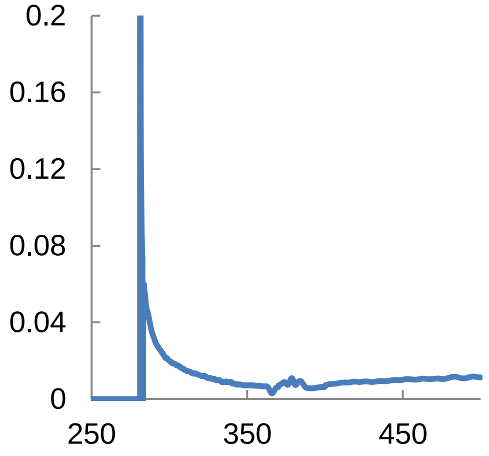

Figure S10. UV–vis absorption spectrum of the solution obtained after 3 days immersion of γ-CD–C_{60} nonwovens in toluene. No peak related on C_{60} was observed.

9. (Figure S11) XRD patterns of β-CD–C_{60}, γ-CD–C_{70}, and gelatin/γ-CD–C_{60} nonwovens prepared by electrospinning

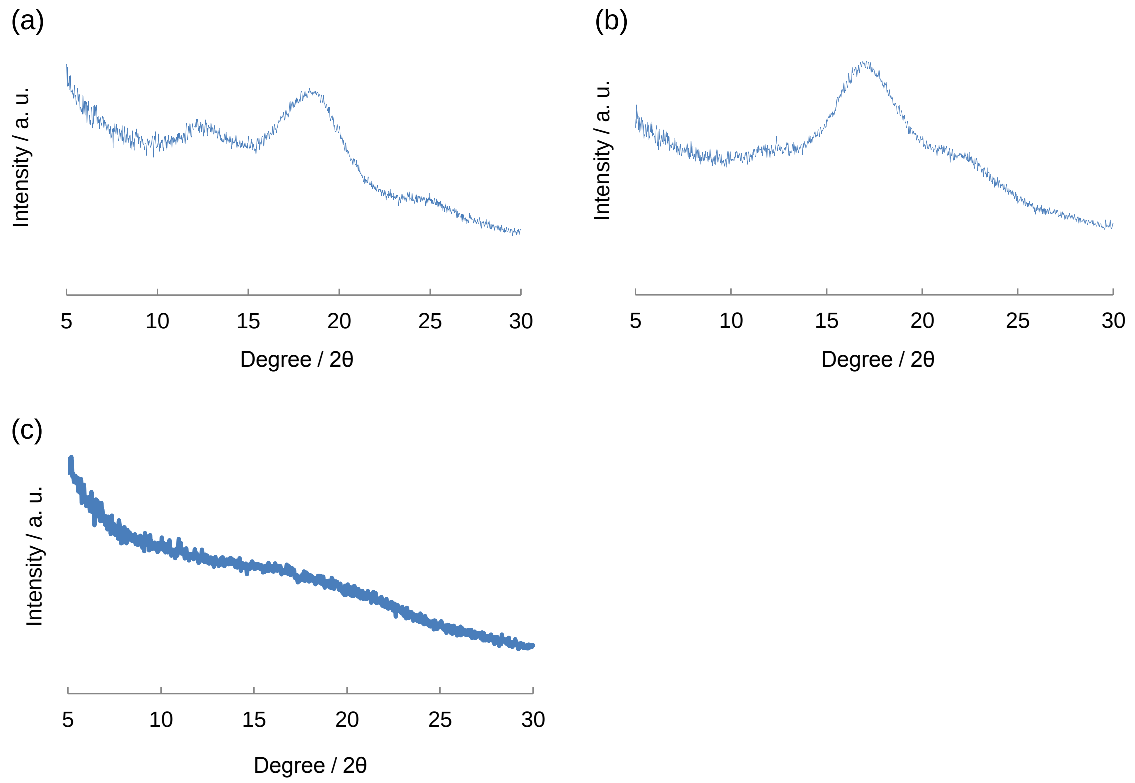

Figure S11. XRD patterns of nonwovens prepared by electrospinning of (a) β-CD–C_{60}, (b) γ-CD–C_{70}, and (c) mixture of gelatin and γ-CD.
